# Empowerment, Pain Control, and Quality of Life Improvement in Early Triple-Negative Breast Cancer Patients through Pain Neuroscience Education: A Prospective Cohort Pilot Study Protocol (EMPOWER Trial)

**DOI:** 10.3390/jpm14070711

**Published:** 2024-07-01

**Authors:** Paola Tiberio, Marco Balordi, Matteo Castaldo, Alessandro Viganò, Flavia Jacobs, Chiara Benvenuti, Rosalba Torrisi, Alberto Zambelli, Armando Santoro, Rita De Sanctis

**Affiliations:** 1 Medical Oncology and Hematology Unit, IRCCS Humanitas Research Hospital, 20089 Milan, Italy; paola.tiberio@cancercenter.humanitas.it (P.T.); flavia.jacobs@cancercenter.humanitas.it (F.J.); chiara.benvenuti@cancercenter.humanitas.it (C.B.); rosalba.torrisi@cancercenter.humanitas.it (R.T.); alberto.zambelli@hunimed.eu (A.Z.); armando.santoro@cancercenter.humanitas.it (A.S.); 2 Department of Biomedical Sciences, Humanitas University, 20072 Pieve Emanuele, Italy; marco.balordi@st.hunimed.eu (M.B.); matteo.castaldo@poliambulatoriofisiocenter.com (M.C.); 3 Department of Health Science and Technology, Center for Pain and Neuroplasticity (CNAP), School of Medicine, Sensory Motor Interaction (SMI), Aalborg University, 9220 Aalborg, Denmark; 4 Clinical Psychology, Clinical Psychophysiology and Clinical Neuropsychology Labs, Parma University, 43126 Parma, Italy; 5 IRCCS Fondazione Don Carlo Gnocchi, 20148 Milan, Italy; avigano@dongnocchi.it

**Keywords:** pain neuroscience education, triple-negative breast cancer, pain, biomedical education, neuropathy, migraine, quality of life, painful syndromes, disability

## Abstract

The treatment of early triple-negative breast cancer (eTNBC) has improved patients’ prognosis but often leads to adverse events and sequelae affecting quality of life (QoL). Pain Neuroscience Education (PNE) is a promising non-pharmacological intervention in this field. Preliminary data have shown the beneficial effect of PNE in BC survivors. However, there are still gaps in knowledge regarding its optimal use in eTNBC. To address this issue, a prospective pilot study will enroll 30 consecutive patients diagnosed with eTNBC at IRCCS Humanitas Research Hospital. The PNE program will consist of 10 weekly sessions to be started within 4 weeks of the onset or worsening of a pain syndrome (PS). QoL, pain, and disability will be assessed before, during, at the end of, and 6 months after PNE using validated questionnaires. Peripheral venous blood samples will be taken before and at the end of PNE to evaluate inflammatory serum biomarker levels. The primary objective is to evaluate whether PNE leads to clinical improvement in QoL and pain. If successful, it will be validated in a larger multi-centric cohort, potentially leading to its widespread implementation as a standard pain management tool for eTNBC patients.

## 1. Introduction

Scientific advancements in oncology have significantly improved the prognosis of early-stage breast cancer (BC) patients through the integration of locoregional (i.e., surgery and radiotherapy) and pharmacological treatments [1]. On the other hand, these treatments increase the risk of sequelae and complications, both short- and long-term, with a significant impact on patients’ quality of life (QoL) [2,3].

Premenopausal women are more likely to develop triple-negative BC (TNBC) [4], though the incidence might vary based on a number of factors, including genetic susceptibility, family history, and lifestyle [5]. Since TNBC is more frequently diagnosed in young, professionally and socially active women, they are generally more inclined to participate in educational therapies aimed at improving their well-being [6]. Regarding side effects, patients with TNBC are more likely to be exposed to more extensive breast surgery (i.e., in the case of BRCA pathogenic variants) and perioperative chemotherapy treatments (neoadjuvant and/or adjuvant) containing anthracyclines and taxanes, which are associated with a high incidence of painful syndromes (PSs) [7].

PSs are the most common comorbid conditions in oncology, greatly impacting patients’ QoL [2,3]. These syndromes, including direct tumor-related pain or pain resulting from treatments, can be multifaceted and can also worsen pre-existing conditions, such as headaches, which are common in younger female patients [8]. Therefore, managing these syndromes is a significant issue for BC patients, especially those with TNBC.

Persistent postoperative pain, a form of neuropathic pain, is a common post-treatment PS, affecting 11 to 57% of patients and often lasting more than three months after surgery [9], especially in TNBC patients, who often require mastectomies and the use of chemotherapeutic agents (such as taxanes and carboplatin) in their therapeutic algorithm, with a notable incidence of post-treatment PSs [10,11]. These agents can cause peripheral sensory neuropathy, particularly in the extremities, which may persist beyond the end of treatment. The incidence of taxane-related neuropathy varies between 30% and 97%, with a persistence rate of up to 40–50% six years after treatment [7]. This neuropathy often manifests as numbness, tingling, and weakness, impairing patients’ daily activities and QoL. Neuropathy can also lead to difficulties with balance and coordination, increasing the risk of falls and injuries [12].

The mechanisms behind PSs in BC are unclear, but research indicates that they may arise from dysregulation of the immune system and neurotransmitters. Cancer is associated with systemic inflammation, and many of its symptoms, including pain, can be attributed to the action of inflammatory cytokines released during the inflammatory process on the peripheral and central nervous systems [13]. In healthy individuals, there is a balance between pro- and anti-inflammatory cytokines, but in cancer patients, this balance is often disrupted. Recent studies have shown that pro-inflammatory cytokines play a significant role in mediating cancer pain by releasing mediators that sensitize nearby nociceptors to painful stimuli and evoke central responses. Some inflammatory markers, such as C-reactive protein (CRP) and erythrocyte sedimentation rate (ESR), are elevated in BC patients and are associated with a worse prognosis [14,15,16]. Moreover, serum levels of certain pro-inflammatory cytokines have been proportionally correlated with the severity of BC-related pain [17]. Lastly, the neutrophil-to-lymphocyte ratio (NLR) is considered a potential prognostic biomarker suitable for assessing the extent of systemic inflammation, contributing to the development of pain and BC progression [18].

Headaches, particularly migraine, are a common pre-existing PS among BC patients. A recent study of 440 patients showed that the prevalence of migraine in BC patients is much higher than expected for the same age group in the general population. Furthermore, this study showed that BC occurs earlier in patients with migraine and that locoregional treatments (especially radiotherapy) can exacerbate the number of headache days in patients with this pre-existing condition. Finally, patients with migraine and tension-type headaches have a higher risk of developing taxane-induced neuropathy. [8]. The Calcitonin Gene-Related Peptide (CGRP), a pro-inflammatory neurotransmitter, plays a significant role in the pathophysiology of migraine and has been found to be increased in migraine patients. Inhibiting CGRP has shown promise in managing migraine pain, and intriguingly, CGRP is also implicated in BC, influencing cell proliferation, differentiation, and angiogenesis [19,20]. It has a U-shaped effect on BC cells, with inhibitory actions at low concentrations and activating effects at high concentrations [21].

There is emerging research into the potential impact of non-pharmacological treatments on BC comorbid conditions. Among these non-pharmacological interventions, Pain Neuroscience Education (PNE) appears promising. PNE aims to help patients understand pain in its full context, emphasizing its emotional, cultural, psychological, and social aspects. The goal is not just to reduce pain but also to improve patients’ physical, emotional, and social functioning. PNE, recognized by the World Health Organization, includes educating patients about pain mechanisms, enhancing coping strategies, and improving the QoL of patients with persistent pain [22].

PNE is aimed at changing patients’ beliefs about pain and its origins, functions, biological processes, and perception-altering factors. The intention is to help patients move away from the purely biomedical perception that pain equals damage and understand it as a multidimensional experience influenced by a network of various central nervous system areas. PNE has been extensively studied in patients with different forms of chronic pain and shown to have positive effects on pain, disability, catastrophizing, and physical performance [23,24].

Preliminary data also suggest that PNE can be useful in patients undergoing surgery for BC. The first pain education programs studied for BC patients were mainly digital. One study showed patient satisfaction and an association between the intervention and a shorter duration of opioid use after surgery, though no significant improvement in pain-related disability, pain intensity, and physical or emotional functioning was observed [25]. Another study supported a personalized eHealth intervention that improved pain-related functioning, physical functioning, and QoL in women with persistent pain after BC treatment. However, the optimal program and mode of PNE administration for these patients are yet to be established [26]. A meta-analysis investigating the effect of patient education in BC survivors reported a short-term improvement in overall QoL, emotional health, and fatigue but a non-significant decrease in pain severity [27].

Overall, the pharmacological management of PSs remains unsatisfactory for patients with TNBC, partly due to oncologic treatments that may intensify these syndromes. In this study, we plan to examine the effectiveness of a program that combines PNE and cognitive training. Although PNE has been effective for many chronic pain conditions, its use has not been tested in cohorts of TNBC patients. We anticipate that it will enhance coping strategies, reduce disability, and improve patients’ QoL.

## 2. Materials and Methods

### 2.1. Study Design

This mono-centric, observational, prospective, pilot cohort study is being led by IRCCS Humanitas Research Hospital in Rozzano, Italy. The study was approved by the IRCCS Humanitas Research Hospital Ethics Committee (protocol identification number: ONC/OSS-17/2023, v.1.1). All patients will sign an informed consent form in accordance with the Declaration of Helsinki.

Our study protocol is presented according to the SPIRIT (Standard Protocol Items: Recommendations for Interventional Trials) checklist (File S1) [28]. 

### 2.2. Participants

This pilot study will enroll patients diagnosed with early-stage TNBC at the IRCCS Humanitas Research Hospital. They will be given a web link to complete a monthly pain questionnaire. Those with a Numeric Rating Scale (NRS) ≥4 will be invited to join the study within 4 weeks of the onset or worsening of the pain syndrome. 

Specifically, the inclusion criteria for patients in the study will be the following:

- Age >18 years;
- A willingness to participate in the proposed study;
- Female sex;
- A histologically confirmed diagnosis of TNBC;
- Stages I–III;
- Indicated for neoadjuvant or adjuvant treatment with anthracyclines and taxanes +/− immunotherapy (i.e., pembrolizumab);
- NRS ≥4;
- The ability to provide informed consent according to the International Conference on Harmonization (ICH)—Good Clinical Practices (GCP) and national/local regulations.

The exclusion criteria will be as follows:

- The recent introduction of analgesic therapies for PSs, with the frequent or continuous use of pain therapies (including non-steroidal anti-inflammatory drugs, steroids, opioids) for chronic PSs; patients on any analgesic therapy will be recruited only if the therapy is stable for at least 3 months;
- Patients eligible for other therapeutic regimens (e.g., cyclophosphamide, methotrexate, 5-fluorouracil);
- Patients who have received or are receiving endocrine therapies for oncological or non-oncological reasons (as these can cause PSs and would represent a potential confounding factor);
- Patients with a strong language barrier;
- The diagnosis of a second primary synchronous or metachronous neoplasm (within 5 years prior to the diagnosis of TNBC);
- The diagnosis of a chronic PS secondary to orthopedic, neurological, or rheumatological disease.

Patients will be allowed to modify their pain management strategies, including analgesic medications, as needed. These adjustments will be documented, and such patients will continue to be monitored and included in the study’s analysis, with appropriate statistical adjustments made to account for any changes in their analgesic use, assessed by applying an equianalgesic scale.

### 2.3. Participant Recruitment and Study Procedures

This project will involve the consecutive enrollment of early TNBC patients with the onset or worsening of a PS (NRS ≥4) who meet the inclusion criteria of the study. 

The evaluation of QoL, perceived pain, and disability will be carried out at baseline (T0) and approximately 5 weeks after the start (T1) and at the end (T2) of PNE; finally, an additional assessment will be performed 6 months after the end of PNE. This evaluation will be conducted using validated questionnaires that measure the overall QoL of BC patients (European Organization for Research and Treatment of Cancer Quality of Life Questionnaire, Breast Cancer-Specific 23 [EORTCQLQ-BR23] questionnaire), pain (NRS), migraine and headaches (Headache Impact Test-6 [HIT-6] and MIgraine Disability ASsessment [MIDAS] questionnaires), and anxiety and depression (Hospital Anxiety and Depression Scale [HADS]).

At timepoints T0 and T2, a peripheral venous blood draw will be collected for the execution of a panel of blood chemical tests (CRP, ESR, Interleukin [IL]-6, IL-8, tumor necrosis factor [TNF]-α, and CGRP, as well as NLR calculation).

Our program involves the administration of a 10-session PNE program on a weekly basis (total of 10 weeks of intervention), in addition to the standard of care, according to the level of pain. The PNE sessions will be conducted by a physiotherapist expert in delivering PNE across different chronic pain populations, with more than 10 years of experience. Each session will be delivered in small groups (3–5 people) using a PowerPoint slide presentation and will last approximately one hour. A part of each session will be dedicated to questions and answers and personal discussion about personal experience.

The contents of the 10 sessions will be as follows:

1. The concept of pain and its biological function.
2. Common misconceptions about pain and its origin.
3. The basics of the neurobiology of pain.
4. What factors alter the perception of pain?
5. The concept of the nervous system as a “protomet” and other metaphors.
6. What happens when pain persists and becomes chronic?
7. How can I train my nervous system?
8. The importance of context, emotional aspects, and communication.
9. The importance of stress management.
10. Long-term pain management.

The program was structured starting from the books “*Explain pain*” [29] and “*Explain pain supercharged*” [30,31], written by the leading researchers on the topic of PNE.

To monitor adherence to the program, we will track in-person participation and monitor who downloads the slides of the PowerPoint presentation. Completing the questionnaires at the 3 timepoints will be a further adherence-monitoring tool.

### 2.4. Objectives

Primary objective:

- The primary objective of the project is to evaluate, through the use of validated questionnaires, whether the implementation of a dedicated PNE and cognitive training program can lead to clinical improvement in terms of QoL and pain in patients with early-stage TNBC.

Secondary objectives:

- To assess the impact of PNE on headaches/migraine in patients affected by this condition prior to the start of the program.
- To evaluate the impact of PNE on anxiety/depression symptoms according to the score of the planned scales.
- To investigate whether serum levels of inflammatory biomarkers correlate with PSs.
- To investigate whether serum levels of inflammatory biomarkers before and after PNE treatment correlate with the response to PNE.
- To compare the results at the end of PNE (T2) with those at six months after the end of the program.

### 2.5. QoL Evaluation

The assessment of QoL, perceived pain, and disability in TNBC patients will be carried out at baseline and during and after training (T0, T1, and T2, respectively) using the following questionnaires (Table 1):

- EORTCQLQ-BR23: This questionnaire is derived from the integration of the general questionnaire for cancer patients (EORTC QLQ-C30) with topics specifically relevant to BC. It consists of 23 items grouped into 5 domains that evaluate the side effects of therapy, disorders at the level of the arm subjected to axillary lymph node surgery, disorders at the breast level, the perception of one’s body image, and sexual functioning. The scores are linearly converted into a scale from 0 to 100. For the functional scales (body image, sexuality, and future expectations), higher scores reflect a better condition, while for symptom scales, higher scores represent worse symptom levels [32,33].
- NRS: This one-dimensional intensity scale assesses the symptom of pain in a simple, immediate, and easily reproducible way (score from 0 to 10) [34].
- HIT-6: This test measures the impact of migraines on common daily activities. It was also validated in the Italian population. It consists of 6 questions concerning how often headaches have caused severe pain, how often they have interrupted daily activities, how often they have resulted in the complete interruption of activities with the need to rest, the presence of headache-related fatigue, irritability, and difficulty concentrating [35]. The possible values for each question are expressed through a Likert scale from 1 to 5 (never, rarely, sometimes, very often, and always) with which a numerical score value is associated (6, 8, 10, 11, 13), and therefore, the total score can range from 36 to 78.
- MIDAS: This questionnaire is widely used to measure the impact of headaches on the individual’s ability to be efficient at work, school, and social activities over the previous 3 months in terms of days of activity lost due to the headache [36]. It consists of 7 items, the first 5 of which evaluate the complete loss of or at least a 50% reduction in productivity (whether work, school, or household). The number of days lost is counted to give the final score. The frequency and intensity of headaches are explored in the last 2 questions. Based on the scores, a disability threshold from 1 to 4 is defined, where the fourth level, the most serious, starts from a score of 21.
- HADS: This scale is used to evaluate the presence and intensity of anxiety and depression symptoms in enrolled patients. This is the most widely used questionnaire for screening for anxiety and depression, validated both in the general population and in patients with a wide range of medical conditions, including patients with BC [37,38]. The scale consists of 14 items divided into two subscales (anxiety and depression) with 7 items each. Each item is assigned a score from 0 to 3, and the total score of each scale is 21 points, where a higher score refers to greater symptomatology. Specifically, a score from 11 to 21 indicates clinically significant cases of anxiety or depression.

**Table 1:** Questionnaires that will be used for the assessment of QoL, perceived pain, and disability in TNBC patients at baseline and during and after training.

Domain	Questionnaire	Description	Scaling	Ref.
QoL	EORTCQLQ-BR23	23 items on side effects, disorders at the axilla and the breast(s), body image, and sexual functioning	From 0 to 100	[32,33]
Pain	NRS	one-dimensional intensity scale	From 0 to 10	[34]
Psychological	HADS	14 items on anxiety and depression	From 0 to 21 (for each 7-point scale)	[37,38]
Headache	HIT-6	6 questions on the impact of migraine on common daily activities	From 36 to 78	[35]
Headache	MIDAS	7 items on the impact of headaches on individual efficiency at work, school, and social activities	From to 28	[36]

### 2.6. Serum Marker Evaluation

At timepoints T0 and T2, peripheral venous blood samples will also be collected to carry out a panel of blood chemistry tests to research biomarkers that may correlate with PSs and/or with the response/non-response to PNE. Specifically, based on the data in the literature, serum levels of CRP, ESR, IL-6, IL-8, TNF-α, and CGRP will be measured. CRP and ESR will be determined according to the standard method currently used at the IRCCS Humanitas Research Hospital clinical analysis laboratory. IL-6, IL-8, TNF-α, and CGRP will be assessed through an enzyme-linked immunosorbent assay (ELISA) following the manufacturer’s instructions, as reported elsewhere [17,39].

### 2.7. Statistical Analysis

#### 2.7.1. Sample Size

The population of the prospective group will consist of 30 consecutive early-stage TNBC patients recruited from our institution’s oncology department. Given the innovative nature of this study, which focuses exclusively on early-stage TNBC patients and utilizes PNE interventions administered by specialized staff, the sample size was determined based on prior prospective studies employing similar interventions [40].

To ensure the representativeness of our results and account for potential dropouts, we have incorporated a margin for sample attrition. Based on typical dropout rates observed in clinical studies, we anticipate a dropout rate of approximately 20%. Therefore, to achieve our target of 30 evaluable patients, we plan to initially recruit 36 patients (30/0.8), ensuring that the final sample size remains robust even if some participants withdraw from the study. This approach will help guarantee that our findings are statistically significant and reflective of the broader population of early-stage TNBC patients undergoing PNE interventions. 

Based on preliminary analyses regarding the study’s primary objective, we will evaluate whether the number of participants should be increased to 50 to identify any factors associated with a better or poor response to the study intervention.

#### 2.7.2. Analysis

Detailed clinical and demographic variables will be recorded for each patient. Frequency tables and descriptive statistics (mean, standard deviation, percentage, median, range) will be used to summarize patient characteristics. 

Both the total score and the score for each domain will be calculated for each questionnaire, according to the nature of the questionnaire. The expected overall clinical improvement will be assessed based on the variation in the total scores of the administered assessment questionnaires at different timepoints (T0, T1, and T2). A positive response to PNE is defined as a 50% or greater improvement in patient-reported outcomes.

Changes in the levels of serum biomarkers between T0 and T2 (baseline and end of training) will be separately analyzed among patients who have responded to PNE (defined as an improvement in patient-reported outcomes of at least 50%) compared to non-responders using a generalized linear model in which the factors involved in the analysis will be the groups (responders/non-responders) and the timepoints (intra-group variable).

As variables of interest and covariates, demographic data, education level, comorbidities, risk factors for BC (e.g., body mass index, cigarette smoking), and menopausal status at diagnosis will be recorded. Additional covariates to be considered include anxiety and depression, which are closely linked to the perception of pain and the experience associated with it.

### 2.8. Data Management

All data obtained in the study described in this protocol will be recorded on electronic case report forms (eCRFs). All data requested on the CRF will be recorded, and any missing data will be explained. If a space is left blank because the procedure was not carried out or the question was not asked, “N/D” will be noted. If the item is not applicable to the individual case, “N/A” will be noted. The CRFs will be dated, completed chronologically, and updated regularly in order to reflect the most recent data on the patients included in the study. All the collected and updated laboratory data will be kept together in a folder in the laboratory. All copies of the laboratory and clinical data will be kept and updated with the same modalities in digital format (eCRF). The Principal Investigator is responsible for ensuring that the data entered into eCRFs are complete and accurate and that entries and updates are performed in a timely manner. Data regarding sample identity, treatment, and processing will also be reported in the laboratory journal, which is permanently located in the laboratory. 

In addition, data from questionnaires will be collected in an anonymous form and kept with the same modalities of laboratory and clinical data.

### 2.9. Data Ownership and Dissemination Policy

According to the IRCCS Humanitas Research Hospital Guidelines on Good Clinical Practice, the sponsor of the study (the institution, should the investigator or study coordinator act as a sponsor in the performance of her/his institutional duties under the employment or collaboration agreement with IRCCS Humanitas Research Hospital) is the owner of the data resulting therefrom. All investigators participating in the study should be made aware of this circumstance and instructed not to disseminate information or data without the institution’s prior express consent. 

Nevertheless, according to the IRCCS Humanitas Research Hospital Guidelines, to grant public access to the full dataset, the database derived from the study will be uploaded to the Zenodo repository (https://zenodo.org/). 

After the completion of the study, the project coordinator will prepare a draft manuscript containing the final results of the study on the basis of the statistical analysis. The manuscript will be distributed to the co-authors for comments and, after revision, will be sent to a major scientific journal. All publications, abstracts, presentations, manuscripts, and slides, including data from the present study, will be submitted to and reviewed by the study coordinator for coordination and homogeneity purposes. In addition, data will be shared with the patients’ foundation “Sorrisi in Rosa by Pink Union” in order to promote a sensitization and awareness campaign on BC-related pain management.

## 3. Discussion

In this study, we plan to examine the activity of a program that combines PNE and cognitive training. We anticipate that it will enhance coping strategies, reduce disability, and improve patients’ QoL.

Thus, the present study will improve the knowledge on the feasibility of administering a PNE protocol to BC patients and, specifically, to early TNBC patients, providing data on the efficacy of PNE in reducing pain and improving patients’ QoL. To our knowledge, this study is the first to test a PNE intervention in early TNBC patients under treatment. To date, only a few clinical trials have investigated the potential efficacy of PNE in reducing chronic postoperative pain in BC patients, with conflicting results [40,41,42,43]. Among the completed trials, only the study by Manfuku and colleagues demonstrated a statistically significant effect of PNE in decreasing pain [43]. Possible biases in the selection criteria could explain the lack of reliability among study findings, for example, the inclusion of patients experiencing low pain intensity. To overcome this issue, we will include only BC patients who experience at least a moderate level of pain (i.e., NRS ≥4). This issue was also addressed in the PaiNEd trial, in which the research group added an inclusion criterion of a visual analog scale ≥ 4 in regions related to the tumor area [41]. 

The existing data suggest that the most effective PNE program for BC patients should be administered by an experienced professional and last longer than current models. However, there is still a knowledge gap in optimizing this therapeutic approach. Undergoing cancer-related therapies is often overwhelming for BC patients. As a result, they often neglect the “side” aspects of their disease due to their busy schedules and main focus on the cancer diagnosis and cure, even in the early setting [8]. Addressing these issues by exploring additional management strategies is crucial. In this context, the implementation of a PNE program for early TNBC patients could be very interesting, as these women are often young and active patients who might be more receptive to educational interventions aimed at promoting their overall well-being and active role in society.

The project outcomes will directly benefit the target population by providing innovative pain management strategies through an exploratory, personalized PNE program delivered by a physiotherapist with experience in PNE programs across different chronic pain populations. The project will also provide medical oncologists with a standardized approach to improving QoL, pain management, and, eventually, patient outcomes. If the project’s outcomes are reached, they will be validated in a larger multi-centric randomized trial, leading to the potential widespread implementation of the PNE program as a standard pain management tool to be integrated into the therapeutic approach for early TNBC patients.

Finally, due to its flexibility, PNE could be extended to other subtypes of BC and to chronic conditions associated with a high incidence of complications and sequelae.

## Data Availability

No new data were created or analyzed in this study. Data sharing is not applicable to this article.

## Supplementary Materials

The following supporting information can be downloaded at https://www.mdpi.com/article/10.3390/jpm14070711/s1: File S1: SPIRIT checklist.
